# The Accuracy of Reporting of the Hypertensive Disorders of Pregnancy in Population Health Data

**DOI:** 10.1080/10641950701826695

**Published:** 2008-08-11

**Authors:** Christine L. Roberts, Jane C. Bell, Jane B. Ford, Ruth M. Hadfield, Charles S. Algert, Jonathan M. Morris

**Affiliations:** 1 Clinical and Population Perinatal Health Research, The Kolling Institute of Medical Research, University of Sydney, Australia; 2 Department of Obstetrics and Gynaecology, Royal North Shore Hospital, St. Leonards, Australia

**Keywords:** Pregnancy hypertension, Preeclampsia, Sensitivity and specificity, Medical records, Validity (epidemiology), International Classification of Diseases

## Abstract

**Objective:**

To assess the accuracy of hypertensive disorders of pregnancy reporting in birth and hospital discharge data compared with data abstracted from medical records.

**Methods:**

Data from a validation study of 1200 women provided the ‘gold standard’ for hypertension status. The validation data were linked to both hospital discharge and birth databases. Hypertension could be reported in one, both, or neither database.

**Results:**

Of the 1184 records available for review, 8.3% of women had pregnancy-related hypertension and 1.3% had chronic hypertension. Reporting sensitivities ranged from 23% to 99% and specificities from 96% to 100%. Using broad rather than specific categories of hypertension and more than one source to identify hypertension improved case ascertainment. Women with severe preeclampsia or adverse outcomes were more likely to have their pregnancy-related hypertension reported. When the hypertension reporting was discordant on the birth and hospital discharge data, the hospital data were more accurate.

**Conclusions:**

Pregnancy-related hypertension is reported with a reasonable level of accuracy, but chronic hypertension is markedly under-ascertained, even when cases were identified from more than one source. Milder forms of hypertension are more likely to go unreported. Studies utilizing population health data may overestimate the proportion of more severe forms of disease and any risk these conditions contribute to other outcomes.

## Introduction

Administrative or population health data sets (PHDS)—such as birth, hospital discharge, pharmaceutical, and registry data—are being used increasingly to evaluate health outcomes and health services (1–12). The population coverage and availability of routinely collected PHDS make them an attractive and inexpensive resource for research, allowing description of the total burden of disease and interventions in the population, assessment of risk factors and causal pathways, and investigation of rare outcomes (13,14). However, there are limitations relating to the completeness and validity of data in studies utilizing single datasets and potential misclassification of exposures and outcomes (13,14). Linkage of PHDS can reduce the problem of under-ascertainment if information is collected on more than one dataset, but this allows the possibility of discrepant or discordant case reports (15–17). Although it is has also been suggested that more severe conditions are likely to be reported in PHDS, we are not aware of any studies that support this conjecture (18).

Rates of the hypertensive disorders of pregnancy from PHDS range from 0.6% to 2.7% for chronic hypertension, 1.5% to 7.7% for preeclampsia, 4.2% to 7.9% for gestational hypertension, and 2.7/10 000 to 6.4/10 000 for eclampsia (7–12). Although the reporting of hypertension in PHDS has been included in several studies in general (19–21), high risk (22), and pregnant inpatient populations (15,23–27) only one focussed specifically on hypertension reporting (27). To our knowledge there are no published reports that validate discordant case reports in linked population health data where cases are identified on more than one data set, or assess the association between disease severity and reporting. In addition, few studies have compared broad (e.g., pregnancy hypertension) and specific (e.g., preeclampsia) diagnostic categories (19,21,28) or hypertension reported using the more detailed Tenth Revision of the International Classification of Disease (ICD10).

The principal aim of this study was to assess the accuracy of the hypertensive disorders of pregnancy reporting in single and linked population health datasets (birth and hospital discharge) compared with data abstracted from medical records. In addition, we examined the accuracy of broad versus specific categorization of hypertension, determined whether women with severe preeclampsia and poor outcomes were more likely to have their hypertension reported in PHDS than women with less severe pregnancy hypertension and determined which data source was more reliable when the PDHS reports were discordant.

## Methods

New South Wales (NSW) is the most populous Australian state with a population of ~6.8 million and 83,000 births per annum in over 100 hospitals, ranging from small rural hospitals to seven tertiary centers. The population health data were obtained from two NSW Department of Health computerized datasets: the Midwives Data Collection (MDC) and the Admitted Patient Data Collection (APDC). The MDC (referred to as ‘birth data’) is a population-based surveillance system covering all NSW births ≥20 weeks gestation or ≥400 g birthweight, and includes information on maternal characteristics, pregnancy, labor, delivery and infant outcomes. The APDC (referred to as ‘hospital data’) is a census of all NSW inpatient hospital discharges; data are coded from the medical records according to the Tenth revision of the International Classification of Diseases (ICD10) (28). The NSW Department of Health performs record linkage of the two datasets and produces de-identified linked birth and hospital records. Hypertension is reported on both the birth and hospital data. In the birth data, reporting is by check-boxes for chronic hypertension and/or preeclampsia. In the hospital data, hypertension is reported using the six major ICD10 codes for hypertension in pregnancy (O11-O16) and a maximum of 40 diagnoses could be made for each hospital admission. We compared hypertension reporting in a sample of records from these two data collections with information abstracted from the corresponding medical records. All women who gave birth in NSW in 2002 and who had a linked birth-hospital record were eligible for the study.

Medical record data were obtained from a validation study of the reporting of maternal medical and pregnancy conditions. The methods have been described elsewhere (30). Briefly, the records of 1200 women giving birth in 2002 were randomly selected and data were abstracted from their medical records by three clinicians experienced in chart review. To ensure reasonable numbers of cases at hospitals providing maternity care for low-risk mothers, small rural maternity hospitals were over-sampled. Although hospitals are only required to record medical conditions that affect the current admission, data were abstracted on any occurrence of hypertension during the pregnancy. Abstracted data (referred to as ‘validation data’) were entered into an electronic database and merged with data from the linked birth and hospital population health datasets (PHDS) for analysis.

In the validation study, the clinical criterion for chronic hypertension was hypertension (≥140 mm Hg systolic and/or ≥90 mm Hg diastolic) diagnosed before conception or before 20 weeks gestation (31,32). Pregnancy-related hypertension was diagnosed as arising after 20 weeks gestation and included gestational hypertension, preeclampsia, and eclampsia. Preeclampsia was hypertension with onset after 20 weeks gestation with one or more of pro-teinuria, renal insufficiency, liver disease, neurological problems, hematological disturbances, or fetal growth restriction and where there was multi-organ disease requiring urgent delivery this was classified as severe preeclampsia (31–33). Gestational hypertension was defined as hypertension arising after 20 weeks gestation without other criteria of preeclampsia but resulting in antenatal admission, antihypertensives, or induction of labor. Abstracters noted whether a diagnosis was recorded and therefore likely to be coded, or whether the clinical criteria were fulfilled but a diagnosis was not recorded. The study was approved by the NSW Department of Health Ethics Committee.

### Data Analysis

To provide unbiased estimates that are representative of the population, all rates, estimates, and exact binomial confidence intervals (95%CI) were weighted by the inverse of the sampling probabilities. Using the validation data as the ‘gold standard’ for hypertension status, we calculated the sensitivity, specificity, positive predictive value (PPV), and negative predictive value (NPV) of PHDS reporting (34). The sensitivity denotes how completely a data source (e.g., the birth data) identified hypertension compared with the ‘true’ hypertension status as determined by the validation data. The specificity denotes correct ascertainment of the nonhypertensive state. The PPV denotes how accurately the PHDS data source identifies hypertension and NPV for the absence of hypertension. As the PPV is the most efficient and informative validation strategy for low incidence conditions (35), the relative accuracy of the different PHDS was assessed by comparing the PPVs using the test of two proportions and a significance level of 0.05 (36). Agreement between the PHDS (birth and hospital data) and the validation data was determined by calculation of the kappa statistic, which adjusts for agreement that would be observed on the basis of chance. Values greater than 0.75 represent excellent agreement beyond chance, values 0.40 to 0.75 represent good agreement beyond chance, and those below 0.40 represent poor agreement (36).

Pregnancy hypertension was used to explore whether reporting differed by maternal age and parity, and whether more severe disease or poor infant or maternal outcomes influenced reporting in the PHDS. The outcomes examined were preterm birth (<37 weeks gestation) and maternal morbidity (a composite measure of major morbidity based on adverse events and medical procedures) (30). The ‘true’ rate of adverse outcomes among women with pregnancy hypertension was determined using the validation data. Then among women with pregnancy-related hypertension identified by the PHDS, the proportion with validated adverse outcomes was determined and compared with the ‘true’ rate. The test of two proportions was used to assess whether differences were greater than expected (*p* < 0.05) (36).

Finally we examined the impact of using different methods of classifying records when the occurrence or type of hypertension was discordant on the birth and hospital data. We compared the odds ratio (OR) of the association between pregnancy-related hypertension and maternal morbidity in the validation data (the ‘true’ estimate of effect) with four previously reported methods of classifying discordant records (17). The following situations were considered partial agreement: one source reported gestational hypertension and the other preeclampsia; reporting only one part of chronic hypertension with superimposed preeclampsia; unspecified hypertension in the hospital data and a type specified in the birth data; and reporting on only one PHDS (17).

## Results

Of the 1200 records selected, 1184 were available for review. The rates of hypertension as determined by the medical record review, birth data, hospital data, and linked data are presented in Table 1. The birth and hospital data underestimated the rates of hypertension. The use of linked data (hypertension in either birth or hospital data) gave rates closer to those in the validation data. Only 12 (0.14%) women in the validation study had chronic hypertension with superimposed preeclampsia.

**Table 1:** Rates of hypertension in the validation study and population health data sets (PHDS).

		Rates of hypertension by data source (%*)			
		
Type of hypertension	No. of “true” cases	Validation study	Birth data	Hospital data	Either birth or hospital data
Any hypertension	178	9.5	6.2	7.2	8.0
Any Chronic hypertension	25	1.3	0.5	0.6	0.8
Any Pregnancy hypertension	165	8.3	5.7	6.0	7.5
Gestational hypertension	72	6.2		3.8	
Preeclampsia	93	2.1		2.3	

* Percentages are weighted for the sampling probability.

In the validation data, 59 (0.9%) records fulfilled the clinical criteria for severe preeclampsia, and all had a recorded diagnosis of preeclampsia (86%) or pregnancy hypertension (14%). Ten (0.4%) records fulfilled the clinical criteria for chronic hypertension, but no diagnosis was recorded in the medical record and similarly for 7 (0.9%) women with gestational hypertension. Of these, 44% and 9% respectively were reported in one or other PHDS.

Of the medical records reviewed, 642 (56%) were considered less than complete, including 22% without antenatal clinic records and 20% private patients. The rate of chronic hypertension was significantly higher (*p* = 0.03) in those with complete documentation (2.1%) compared with those with incomplete or no antenatal records (0.7%). However, there was no statistically significant difference in the rate of pregnancy hypertension among women with complete versus incomplete records (9.1 % versus 7.7%, *p* = 0.4).

The hypertension reporting characteristics of the PHDS compared with the validation data are shown in Table 2. For the specific types of hypertension, sensitivities ranged from 23% to 85% and specificities from 96% to 100%. Broad categorization of hypertension into any pregnancy-related hypertension or any hypertension, increased the sensitivity with little impact on the specificity, as did identifying cases for either PHDS. Kappa statistics were also highest for the grouped hypertension categories and for hypertension identified on either dataset. Sixteen of the 25 NPVs (data not shown) were over 98.0%, ranging from 96.9% (95%CI, 95.4% to 97.6%) for gestational hypertension to 100% for preeclampsia identified from either dataset. There was a tendency for the hospital data to be more accurate than the birth data, with generally higher PPVs, NPVs and kappa statistics for all the hypertensive disorders. However, with the exception of preeclampsia reporting, the differences were not statistically significant.

**Table 2:** Characteristics of PHDS reporting compar ed with the true occurrence of disease as abstracted from the medical record.

	Reporting characteristics by PHDS source % (95% confidence interval)*											
	
	Birth data				Hospital data				Either birth or hospital data			
			
Hypertension type	Sensitivity	Specificity	PPV	κ	Sensitivity	Specificity	PPV	κ	Sensitivity	Specificity	PPV	κ
Chronic hypertension	22.6 (5.7–50.7)	99.8 (99.3–100)	56.3 (15.7–91.2)	0.32	44.4 (19.7–71.3)	100 (99.7–100)	100 (57.9–100)	0.61	46.9 (21.6–73.4)	99.8 (99.3–100)	72.7 (36.9–94.7)	0.57
Gestational hypertension	—	—	—	—	47.8 (36.0–59.8)	99.2 (98.4–99.6)	78.9 (64.1–89.7)	0.58	—	—	—	—
Preeclampsia	84.7 (64.8–95.8)	96.0 (94.7–97.1)	31.6 (20.8–44.0)	0.44	71.0 (49.7–87.1)	99.2 (98.5–99.6)	66.7 (46.0–83.5)	0.68	99.1 (84.8–100)	95.8 (94.4–96.9)	33.8 (23.2–45.7)	0.49
Any Pregnancy hypertension	63.3 (53.0–72.8)	99.5 (98.9–99.8)	92.4 (83.3–97.4)	0.73	68.2 (58.1–77.2)	99.6 (99.1–99.9)	94.4 (86.2–98.4)	0.78	82.3(73.3–89.3)	99.3(98.7–99.7)	91.9(84.1–96.6)	0.86
Age <35 years	67.7 (56.3–77.7)	99.4 (98.7–99.8)	91.5 (81.3–97.1)	0.76	74.7 (63.7–83.7)	99.6 (98.9–99.9)	93.9 (84.9–98.4)	0.82	88.8 (79.8–94.8)	99.2 (98.4–99.7)	91.1 (82.4–96.3)	0.89
Age ≥35 years	44.6 (21.9–69.1)	100 (98.1–100)	99.1 (62.9–100)	0.59	40.2 (18.5–65.1)	99.9 (98.0–100)	98.3 (58.9–100)	0.55	54.0 (29.7–77.0)	99.9 (97.9–100)	98.0 (66.5–100)	0.68
Primiparous	72.9 (57.5–85.1)	100 (99.1–100)	99.8 (89.0–100)	0.83	80.1 (65.5–90.5)	100 (99.1–100)	99.8 (89.8–100)	0.88	92.6 (80.7–98.3)	100 (99.1–100)	99.7 (90.9–100)	0.96
Multiparous	55.4 (41.3–69.0)	99.2 (98.2–99.7)	85.5 (69.4–95.0)	0.65	58.3 (44.1–71.6)	99.4 (98.5–99.8)	88.9 (73.8–96.9)	0.69	73.8 (60.0–84.8)	98.9 (97.8–99.6)	84.9 (71.5–93.7)	0.77
Any hypertension	65.0 (55.4–73.7)	100 (99.6–100)	99.8 (94.7–100)	0.77	73.8 (64.7–81.7)	99.8 (99.3–100)	97.6 (91.7–99.7)	0.83	81.4 (73.0–88.1)	99.8 (99.3–100)	97.7 (92.2–99.7)	0.88

* % are weighted for the sampling probability.

PPV = Positive Predictive Value

κ = Kappa statistic

Table 2 also shows the pregnancy hypertension reporting characteristics of the PHDS compared with the validation data stratified by maternal age and parity. Among the study population 18.2% of women were aged ≥35 years, and 39.9% were having their first baby. The reporting sensitivities and kappas were higher for women aged <35 years but the PPVs were lower, and the differences in reporting were not statistically significant. In contrast, the PPV and NPV were significantly higher for primiparous women than for multiparous women when pregnancy hypertension was identified in either dataset.

In the validation data, 2 women (1.5/10,000) had eclampsia and while both were identified in the hospital data, there were also 6 false-positives (2 with preeclampsia and 4 with gestational hypertension but no record of convulsions) giving a PPV of only 23.5%.

All women identified as having severe preeclampsia in the validation data were reported with pregnancy hypertension in the hospital data and 87% were reported in the birth data, higher than the overall sensitivities for pregnancy hypertension (68% and 63% respectively); under-reporting was concentrated among the less severe forms of pregnancy hypertension. Thus the PHDS included a higher proportion of women with severe preeclampsia than the ‘true’ rate in the validation data, although the differences were not statistically significant (Table 3). Women with a preterm birth and/or maternal morbidity were also more likely to have their pregnancy hypertension reported in the PHDS (Table 3). Restricting analyses to women with pregnancy hypertension reported in both PHDS datasets resulted in an over-representation of severe disease (as indicated by the highest rates of adverse outcomes) and under-representation of milder disease or women who were well managed. Accepting a report of pregnancy-related hypertension from either data set identified the greatest number of women with pregnancy-related hypertension and had rates of adverse outcomes closest to the ‘true’ rates.

**Table 3:** Rates of validated adverse outcomes by source of pregnancy hypertension report.

		Validated outcomes %*		
		
Data source	Women with pregnancy-related hypertension,†	Severe preeclampsia	Preterm <37 weeks	Maternal morbidity
*“True” pregnancy hypertension*‡	*165*	*10.6*	*9.0*	*4.9*
Hypertension Reporting Source				
Birth data alone	109	14.5	13.0	6.5
Hospital data alone	142	15.5	13.2	6.9
Either hospital or birth data	157	12.8	11.0	5.7
Both hospital and birth data	94	18.7	16.7	8.4

* Weighted percentage of validated adverse outcomes among women with pregnancy hypertension.

† Number of women in the validation study with pregnancy-related hypertension according to the data source.

‡ ‘True’ rates of adverse outcomes among women with any pregnancy-related hypertension, according to the medical records. The subsequent rows show that those with severe disease or poor outcomes are more likely to be reported in PHDS but the differences were not statistically significant (*p*> 0.1).

Finally, we examined the 100 (6%) records where the PHDS had discordant coding of hypertension including 3% where the hypertension was reported in only one dataset. The hospital data included 9 (0.7%) with ‘unspecified maternal hypertension’, and the majority of these (98.5%) were identified as gestational hypertension in the validation study. Overall, the hospital data were more reliable; of the discordant records, the hospital data were correct or partially correct for 76% compared with only 22% for the birth data (*p* < 0.05). Using the four published methods of classifying discordant records (17) gave odds ratios (OR) for pregnancy-related hypertension and maternal morbidity that ranged from 3.1 to 4.7 compared with the ‘true’ estimate of 3.4 (95%CI, 1.2 to 9.5) although the confidence intervals of all estimates overlapped. Restricting the analysis to records with perfect agreement on hypertension status gave the most extreme odds ratio (4.7; 95%CI, 1.2 to 17.8) while including perfect and partial agreement gave an estimate that was closest to the ‘truth’ (3.2; 95%CI, 1.0 to 10.8).

## Discussion

This study reports the accuracy of PHDS in identifying the true prevalence of the hypertensive disorders of pregnancy and is a validation study as opposed to an audit of coding practices in which experienced coders independently reassign ICD codes to a sample of hospital records (19). We have demonstrated for the first time that the reporting of hypertension in pregnancy varies according to the presence of risk factors and adverse outcomes and that hospital discharge data should be used in preference to birth data when discrepancies between the two data sources occur. Simultaneously validating two datasets allowed the assessment of discordance between the datasets. We also found that using broad rather than specific categories of hypertension and more than one source to identify hypertension improved case ascertainment. Quantification of under-reporting allows the adjustment of prevalence estimates (38).

Although it has been previously demonstrated that reliance on single PHDS is likely to result in under-reporting of disease prevalence, our findings suggest that under-reporting is not random. Not only were women with severe preeclampsia more likely to be reported than those with milder forms of pregnancy hypertension, so were those with adverse maternal and infant outcomes. Although the validation sample had insufficient power to demonstrate a statistically significant association between severity of outcome and reporting, there was a consistent tendency for disproportionate reporting in the PHDS of women with adverse outcomes. This finding is unlikely to be explained by insufficient diagnostic fields (20), as there were 40 diagnosis fields available in our hospital data and the maximum number used in this sample was 16. The women whose diagnoses were most likely to remain unreported were those with less severe disease or, perhaps more importantly for health services research, those who were well managed and did not suffer an adverse outcome. We also found that primiparous women were significantly more likely to have their pregnancy hypertension reported than multiparous women, which could be related to higher rates of preeclampsia and adverse outcomes in first pregnancies (8). We have previously shown that reporting in PHDS varies by mode of delivery (38). The association between reporting, risk factors and adverse out-comes needs to be examined for other conditions and in other populations.

For rare outcomes such as eclampsia, in which fewer than 1% of the women have the condition, the number of false-positives can outweigh the number of true positives resulting in a low PPV (<50%). Analyses relying on conditions with low PPVs would include more false-positive reports than true cases. The reporting of eclampsia has previously been identified as inaccurate with PPVs of 6 to 50%, and should not be relied upon (15,27,39).

In audits of general hospital populations using ICD8, ICD9, and ICD10, broader diagnostic categories have been found to be more reliable than individual diagnoses (19,21,28). As the International Classification of Diseases does not provide clinical definitions of the hypertensive disorders of pregnancy, we found considerable misclassification of gestational hypertension and preeclampsia in both data sets and collapsing these categories into a single pregnancy hypertension category improved the PPV. Further improvements in the PPV were obtained by aggregating the pregnancy and chronic hypertension categories to ‘any hypertension.’ However, an ‘any hypertension’ variable may have limited clinical utility because chronic and pregnancy hypertension have different risk factors, care requirements and adverse event probabilities (8,31,32).

Although record linkage improves case identification when conditions are reported on more than one dataset, the researcher may be faced with discordant diagnoses from different data sources (17). We have identified that half the hypertension reporting discrepancies were due to under-reporting on one dataset. Of the remaining discordant reports, over 99% involved misclassification of gestational hypertension and preeclampsia or partial reporting of preeclampsia superimposed on chronic hypertension. In general, the hospital discharge data were more reliable, and should be used in preference to birth data collections. For specific research questions, the preferred method for selecting cases of hypertension from PHDS might depend upon whether hypertension is being examined as a risk factor or an outcome. If used as a risk factor, it will be desirable to minimize under-reporting and include cases identified from any data source. However, if preeclampsia is being studied as an outcome of pregnancy, it might be preferable to identify confidently the severe manifestations that adversely affect the pregnancy. Linked hospital and birth data are useful for investigating major maternal morbidity as serious conditions are generally well reported (30).

It was not surprising that chronic hypertension was under-reported in both data sources. In this study, data were abstracted on the basis of any occurrence of hypertension during pregnancy. In contrast, hospital discharge records are only required to include conditions that affect the current hospital admission, and birth data are more accurate at recording birth events than medical conditions (25). Thirty percent of the women with chronic hypertension fulfilled the clinical criteria but did not have the diagnosis recorded. Some of these records were coded as chronic hypertension in the hospital data indicating that some coding is based on diagnostic criteria or recognizes diagnoses of chronic conditions from previous admissions. Furthermore, the medical chart may be a poor gold standard for chronic hypertension if the records are not complete. There was a significant difference in the prevalence of chronic hypertension among complete and incomplete records and the ‘true’ prevalence of chronic hypertension in pregnancy may be around 2%. Pregnancy-related hypertension was not affected in the same way because pregnancy hypertension was usually an important diagnosis in the birth admission. It is important that clinicians ensure that records are complete and accurately reflect the final diagnoses, risk factors, and outcomes. Longitudinal linkage of population data to antenatal hospitalizations may also allow improved identification of chronic hypertension.

Although this study is based on perinatal data, the findings are likely to be applicable to the linkage of any specialized health datasets that include information on diagnoses or procedures. Increasing linkage of such datasets and additional linkages with population health registries, such as stroke, congenital anomaly and pharmaceuticals (1–12), will increase the need for assessments of the usefulness and accuracy of the linked data.
